# Network analytical investigation of relationships between symptoms of common mental disorders among refugees and asylum seekers in Türkiye

**DOI:** 10.1017/S2045796024000696

**Published:** 2024-11-05

**Authors:** G. Kurt, M. Ekhtiari, A. de Graaff, M. Ersahin, P. Specker, M. Sijbrandij, A. Nickerson, C. Acartürk

**Affiliations:** 1 School of Psychology, University of New South Wales, Sydney, NSW, Australia; 2 Department of Sociology, Koc University, Istanbul, Türkiye; 3 Department of Clinical, Neuro- and Developmental Psychology, WHO Collaborating Center for Research and Dissemination of Psychological Interventions, Amsterdam Public Health Research Institute, Vrije Universiteit Amsterdam, Amsterdam, The Netherlands; 4 Department of Clinical Psychology, Erasmus University Rotterdam, Rotterdam, Netherlands; 5 Department of Psychology, Koc University, Istanbul, Türkiye

**Keywords:** anxiety, comorbidity, depression, mentalhealth, network analysis, posttraumatic stress disorder, refugees

## Abstract

**Aims:**

Forcibly displaced people, such as refugees and asylum-seekers (RAS), are at higher risk of mental disorders, mainly post-traumatic stress disorder (PTSD), depression and anxiety. Little is known about the complex relationships between these mental disorders among culturally and linguistically diverse RAS. To investigate this, the present study applied a novel network analytical approach to examine and compare the central and bridge symptoms within and between PTSD, depression and anxiety among Afghan and Syrian RAS in Türkiye.

**Methods:**

A large-scale online survey study with 785 Afghan and 798 Syrian RAS in Türkiye was conducted in 2021. Symptoms of PTSD (the short form of Post-Traumatic Stress Disorders Checklist [PCL-5]), depression and anxiety (Hopkins Symptoms Checklist-25) [HSCL-25]) were measured via self-administrated validated instruments. We conducted network analysis to identify symptoms that are most strongly connected with other symptoms (central symptoms) and those that connect the symptoms of different disorders (bridge symptoms) in R Studio using the qgraph package.

**Results:**

Overall, Afghans and Syrians differed in terms of network structure, but not in network strength. Results showed that feeling blue, feeling restless and spells of terror or panic were the most central symptoms maintaining the overall symptom structure of common mental disorders among Afghan participants. For Syrian participants, worrying too much, feeling blue and feeling tense were identified as the central symptoms. For both samples, anger and irritability and feeling low in energy acted as a bridge connecting the symptoms of PTSD, depression and anxiety.

**Conclusion:**

The current findings provide insights into the interconnectedness within and between the symptoms of common mental disorders and highlight the key symptoms that can be potential targets for psychological interventions for RAS. Addressing these symptoms may aid in tailoring existing evidence-based interventions and enhance their effectiveness. This contributes to reducing the overall mental health burden and improving well-being in this population.

## Introduction

Forced displacement has become one of the most intricate global challenges in the 21st century. Millions of people are forced to leave their homes due to a myriad of push factors, such as political instability, conflict and climate change. By the end of 2023, the number of forcibly displaced people has exceeded 117 million, of whom over 37 million are refugees and asylum-seekers (RAS). In 2024, the majority of the world’s refugees are from Syria and Afghanistan, the countries facing the most protracted humanitarian crises of all time (United Nations High Commissioner for Refugees, 2024). RAS must contend with a complex web of traumatic experiences and displacement-related stressors, all of which impair their mental health and functioning (Patanè *et al.*, 2022). A recent meta-analysis showed the prevalence of common mental disorders among RAS, namely post-traumatic stress disorder (PTSD), depression and anxiety, at 11%, 31.5% and 31.4%, respectively (Blackmore *et al.*, 2020). These rates are even higher in low- and middle-income countries where the majority of forcibly displaced people live (Patanè *et al.*, 2022). Numerous studies found that the rate of comorbidity between two or more mental disorders can be very high among displaced populations, especially if they are seeking treatment. For example, in a study with outpatient refugees in Switzerland, approximately half (47%) of the sample had both PTSD and depression (Nickerson *et al.*, 2017). Similar rates were observed among RAS in other high-income countries such as Australia (Momartin *et al.*, 2004) and Germany (Nesterko *et al.*, 2020). A recent study among Syrian and Afghan RAS in Türkiye showed that 40–50% of participants suffer from at least two mental disorders and 25–37.7% were likely to be diagnosed with PTSD, depression and anxiety together (Kurt *et al.*, 2022). Comorbidity is an important factor to consider in psychological interventions as it is associated with more severe functional impairment and health outcomes (Momartin *et al.*, 2004; Nickerson *et al.*, 2017). Individuals with comorbid mental disorders are also less likely to benefit from interventions (Amati *et al.*, 2018).

Despite the high rates of comorbid mental disorders among RAS, there is relatively scant information on how distinct mental disorders co-occur. One way to better understand comorbidity is by examining the interconnectedness of symptoms across mental disorders. Network analysis provides a novel approach to investigating these symptom-level associations (Cramer *et al.*, 2010). To date, most studies on refugee mental health considered individual symptoms as distinct, independent entities caused by or reflected by a latent construct of PTSD, depression or anxiety. This approach overlooks the inherently complex relationship between psychological symptoms (Borsboom *et al.*, 2003). As an alternative to this, the network analytical approach posits that mental disorders are manifestations of a network of causal interactions (edges) among individual symptoms (nodes). Rather than being reflected by an underlying latent construct, this approach focuses on uncovering the relationships between the symptoms and identifying where the cycle of interconnectedness can be intervened (Schmittmann *et al.*, 2013). Network analysis is considered a more plausible approach for capturing the relationships among the symptoms and identifying potential targets for treatment, which can yield a series of implications for effective program design and delivery (Rodebaugh *et al.*, 2018). According to network analysis, comorbidity occurs as a result of interactions between symptoms of distinct mental disorders (Cramer *et al.*, 2010). Two clusters of symptoms are key to understanding this symptom interconnectedness: central symptoms and bridge symptoms. Central symptoms are those with overall more connections to the other symptoms within the network, playing a key role in maintaining the overall structure of the symptom network. Bridge symptoms, on the other hand, refer to the symptoms connecting one disorder to another, therefore driving the co-occurrence of comorbid mental disorders. Identification of both central and bridge symptoms provides insights into the interconnectedness within and between the symptoms of mental disorders, elucidating the underlying structure of comorbidity. These symptoms constitute key targets of effective psychological interventions.

Applying the network approach to refugee experiences and mental health has not been common until recently. A recent study conducted in Kenya focused on the identification of the central symptoms of PTSD such as concentration problems and emotional numbing among culturally diverse refugee communities (Kangaslampi *et al.*, 2021). In another study among treatment-seeking RAS (Spiller *et al.*, 2017), emotional cue reactivity was found to be the central symptom of PTSD. Other studies further examined the interconnectedness among post-displacement stressors (Wicki *et al.*, 2021), the relationship between various conflict and displacement stressors, symptoms of depression and PTSD (Behrendt *et al.*, 2023; De Schryver *et al.*, 2015) and stress-related hormones (de Graaff *et al.*, 2024). Only a few studies investigated the interconnectedness between the symptoms of comorbid mental disorders among RAS with evidence from those resettled in high-income settings (Schlechter, Hellmann, et al. 2021; Schlechter, Wilkinson, et al., 2021). While these studies demonstrated central symptoms (e.g., feeling tense or worthlessness) maintaining the network of trauma-related mental disorders such as depression, anxiety and somatization, none of them focused on unpacking the key symptoms responsible for the interconnectedness among the common mental health problems among RAS in the first place.

Considering the array of social and economic ramifications related to mental health problems (McDaid and Park, 2023), prevention and treatment of these problems have become a pressing public health issue in refugee-receiving countries. High mental health burden and low treatment utilization among forcibly displaced populations have led to a proliferation of studies focusing on designing and testing scalable psychological interventions. So far, numerous psychological interventions based on evidence-based transdiagnostic strategies have been developed and culturally adapted for RAS in various settings (Bryant, 2023). Most of the interventions included multiple evidence-based components such as psychoeducation, emotion regulation, problem management and behavioral activation (Schäfer *et al.*, 2023). To further refine and improve the effectiveness of these interventions, they can be tailored to be more specific and targeted to address the key symptoms, effectuating change in the symptoms network. To do so, it is imperative to identify the main symptoms and mechanisms responsible for comorbid mental disorders.

The present study aimed to unpack and compare the intricate relationships between the symptoms of highly prevalent mental disorders, PTSD, depression and anxiety among Afghan and Syrian displaced people in Türkiye to guide the development and implementation of effective psychological interventions. By utilizing a network analytical approach, we aimed to identify the central and bridge symptoms of PTSD, depression and anxiety, leading to high rates of comorbidity. Given the heterogeneity in experiences and mental health of linguistically and culturally diverse forcibly displaced groups (Blackmore *et al.*, 2020), we intended to uncover similarities and differences in how mental health symptoms are interrelated among Afghan and Syrian RAS, respectively. In the present study, we examined the symptom network structure of highly prevalent and comorbid mental disorders (PTSD, depression and anxiety) and tested whether RAS from different backgrounds presented similar symptom networks. Given the scarcity of studies in the literature, we did not posit specific hypotheses as to how the symptoms would interact with each other or how the network structures would differ between Syrian and Afghan RAS.

## Methods

### Participants and study design

Two parallel online survey studies were conducted with Afghan and Syrian forcibly displaced people in Türkiye via local non-governmental organizations and a municipality that works with Afghans and Syrians. The inclusion criteria for this study were (1) being at least 18 years old, (2) being literate in Dari or Arabic and (3) having been forced to leave Afghanistan or Syria due to unrest and flee to Türkiye. We note that Türkiye grants refugee status only to those fleeing persecution or violence from European countries. However, for simplicity, we used the term RAS throughout the study. Participants were asked to fill out the online survey via Qualtrics and were compensated with a grocery voucher (US$3.75) for their time and participation. The ethical approval was obtained by the university’s ethics committee [IRB approval number]. In the Afghan sample, 1027 individuals initially intended to participate in the survey, 877 provided consent and 812 were eligible. Of the 812 eligible participants, 785 completed the survey after passing attention checks, yielding a response rate of approximately 76.44%. In the Syrian sample, the response rate was 77.18%, with 1034 initially intending to participate, 854 providing consent, 841 being eligible and 798 completing the survey.

### Measures

#### Anxiety and depression symptoms

We measured the symptoms of anxiety and depression using the Hopkins Symptom Checklist with 25 items (HSCL-25; 10 items for anxiety and 15 items for depression) rated on a 4-point Likert Scale (1 = not at all, 4 = extremely). Participants were asked to rate how much they are bothered or distressed by the given symptoms (e.g., feeling tense or keyed up for anxiety and feeling low in energy, slowed down for depression) in the last week, including today. Higher scores on items indicate a higher level of psychological distress that participants have experienced in the last week. The scale has been widely used in multiple languages and among several culturally diverse groups including Afghans and Syrians (Acarturk *et al.*, 2021; Wind *et al.*, 2017). The internal consistencies for both groups were excellent in the present study, with a Cronbach’s alpha of *α* = 0.92 for anxiety in both groups and scores of 0.94 for depression among Afghans and *α =* 0.93 for Syrians.

#### PTSD symptoms

The short form of Post-Traumatic Stress Disorders Checklist (PCL-5) based on the DSM-5 diagnostic criteria for PTSD (Zuromski *et al.*, 2019) was used to assess PTSD symptoms among participants. Four items tapping into the domains of hyperarousal, intrusion, avoidance and negative cognition were asked to the participants to indicate to what extent they had experienced these symptoms in the past month on a 5-point Likert Scale (0 = not all, 4 = extremely). Higher scores indicate a higher self-reported level of PTSD symptoms. Both Arabic and Farsi versions of the scale were used in previous studies (e.g., Ibrahim *et al.*, 2018; Koch *et al.*, 2020). The Cronbach’s alpha was *α =* 0.83 for Afghans and *α =* 0.80 for Syrians in the present study.

### Statistical analysis

The statistical analysis and results were reported based on the recommended standards for general reporting for the network analysis (Burger *et al.*, 2023, Burger et al.,^2020^). The results are based on the standardized scores.

#### Item selection

Before proceeding with network estimation, we inspected the topological overlap of the items of the scale by examining bivariate correlations between the items and theoretical overlap. Except for HSCL-1 (suddenly scared for no reason) and HSCL-2 (feeling fearful), none of the correlations exceeded 0.60 in both samples. As these two items represent related but distinct emotional reactions (Grillon, 2008), we included both in the final network estimation.

#### Estimating networks and centrality indices

We estimated one network each for Afghan and Syrian participants with 29 variables (symptoms of PTSD, depression and anxiety) using the EBICglasso function in the qgraph package in R Studio. In the network models, each item in the measures is represented by nodes and edges represent regularized partial correlations between nodes. Edge values range from −1 (negative relationship, red color) to +1 (positive relationship, blue color) with thicker edges representing stronger associations (larger regularized partial correlations) between the nodes. Glasso networks with regularized partial correlations calculate the relationship between the variables while taking the effects of other variables into account, help handle a large number of variables and introduce sparsity into the networks. The EBICglasso function also applies regularization in the network to shrink weak or spurious connections between nodes to ensure that only meaningful connections are retained in the final network model. This helps mitigate the potential biases that might arise from the unequal distribution of items across the measures in the present study. We chose Spearman correlations over polychoric correlations as the latter yielded unreliable parameter estimates such as a non-positive definitive correlation matrix. This is likely to happen due to the non-normal distribution of ordinal data and fewer response options in the items, or a relatively small sample size. In such cases, the Spearman correlations can provide more stable and robust estimations (Epskamp and Fried, 2018). The following centrality indices were calculated and standardized in each network model: betweenness (the number of times that one node is in the shortest path between other nodes), closeness (the inverse of the sum of distances from one node to others) and strength (the absolute sum of the edge weights (partial correlations) connected to each node) to identify the most central symptoms (maintaining symptoms) in each network model (McNally, 2016).

#### Network stability

The network stability (internal reliability of networks) was tested using the bootnet package in R Studio. We bootstrapped with 2500 iterations to estimate 95% confidence intervals for edge weight and conducted an edge weight difference test to statistically compare whether edges between different nodes are significantly different. We, then, calculated the centrality stability coefficient to examine the robustness of centrality indices. The correlation stability coefficient (CS-coefficient) is calculated by repeated subsampling of data by dropping a proportion of cases each time and estimating centrality indices for each subsample. The coefficient represents the maximum proportion of cases that can be dropped while still retaining a correlation of 0.70 between the original centrality indices and those based on subsamples in at least 95% of cases. cases. A minimum CS-coefficient of 0.25 is used as a threshold (Epskamp *et al.*, 2018).

#### Bridge symptoms

To identify bridge symptoms among three mental health conditions, we used the bridge function of the networktools package in R Studio to compute bridge centrality statistics (Jones *et al.*, 2021). We defined the clusters of symptoms as different communities in our analysis (community 1 for anxiety symptoms, community 2 for depression symptoms and community 3 for PTSD symptoms). The bridge function uses partial correlations between nodes to calculate bridge centrality statistics which are bridge strength (the sum of the absolute value of all edges between a node in one community and all the other nodes in other communities) and bridge expected influence (one step) (the sum of the value of all edges between a node in one community and all the other nodes in other communities) (Robinaugh *et al.*, 2016). We reported the nodes with the highest bridge strength and bridge expected influence as bridge symptoms across three mental health problems.

#### Network comparisons

At the final stage of our analysis, we conducted network comparison tests to examine whether the network structures and strength of connections between symptoms were significantly different between Afghan and Syrian participants. To control for the effect of gender, we also conducted network comparison test between female and male participants within each group (Syrians and Afghans). We used the Network Comparison Test Package in R Studio (van Borkulo et al., 2023) to estimate the invariance of network structure and global strength. A *p*-value >0.05 for network invariance structure indicates that there is no significant difference between the two comparison groups (Afghans vs. Syrians) in terms of how their symptoms are connected, meaning that the network structures of Afghans and Syrians do not differ in their overall strength of edges. A *p*-value >0.05 for global strength invariance, on the other hand, indicates that the weighted absolute sum of all edges in one network is not significantly different from that of another network, showing similar overall strength of connections between nodes (symptoms).

## Results

### Sample characteristics

785 Afghan (33.8% female) and 798 Syrian (58.5% female) refugees participated in the study. The average age was 29.60 years (SD = 9.50, ranging from 18 to 67) for Afghans and 33 years (SD = 10.62, ranging from 18 to 75) for Syrians. The majority of Syrians were living under temporary protection status (713, 89.3%) while half of the Afghans were asylum-seekers (433, 55.2%), followed by those with temporary residency permits (138, 17.6%). The mean number of years in education was 11.5 (SD = 4.24) years for Afghans and 9.20 (SD = 4.24) years for Syrians. A detailed description of the sample and differences can be found elsewhere (Kurt *et al.*, 2022). The demographic characteristics of both samples were comparable to general Afghan and Syrian population in Türkiye (Presidency of Migration Management, 2023; Eryurt and Koç; 2017).

### Network stability and central symptoms

**Figure 1.**
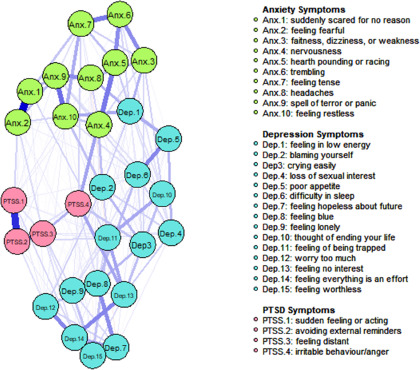
Estimated network model for Afghan sample.

**Figure 2.**
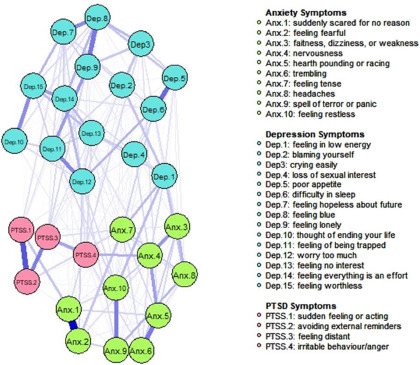
Estimated network model for Syrian sample.

**Figure 3.**
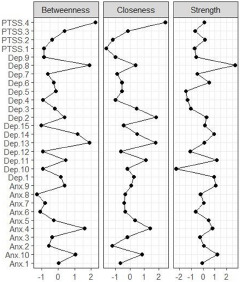
Standardized centrality indices for the Afghan sample.

**Figure 4.**
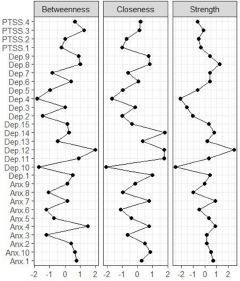
Standardized centrality indices for the Syrian sample.

The network models for Afghans and Syrians are represented in Figs. 1 and 2. Both estimated networks were highly stable. The stability analyses of the networks are presented in Supplementary Material 1. The high overlap between the bootstrapped edge weights and those of the full sample indicated stable network estimation. The CS-coefficient for centrality indices was above the threshold, with strength having the highest correlation of 0.75 in both samples. This was in line with previous research showing that strength has more robust stability and replicability (Epskamp *et al.*, 2018). Thus, our results were based on strength centrality. We also provided closeness and betweenness values of those with the highest strength centrality. In the Afghan sample, the symptoms with the highest strength centrality were feeling blue (Dep.8) (Strength (S) = 2.735) (Closeness (C) = 0.367, Betweenness (B) = 1.903) feeling restless (Anx.10) (S = 1.221) (C = 0.840, B = 1.028) and spells of terror and panic (Anx.9) (S = 1.057) (C = 0.070, B = 0.372). In the Syrian sample, worrying too much (Dep. 12) (S = 2.478) (C = 1.784, B = 2.019), feeling blue (Dep.8) (S = 1.253) (C = 0.820, B = 1.022) and feeling tense (Anx.7) (S = 0.907) (C = 0.769, B = 0.150) had the highest strength centralities. Figures 3 and 4 represent the standardized centrality indices of each symptom for both samples.

### Bridge symptoms

**Figure 5.**
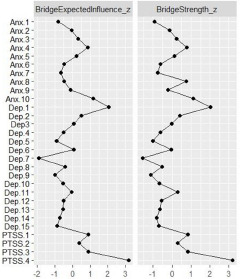
Standardized bridge strength and bridge expected influence for the Afghan sample.

**Figure 6.**
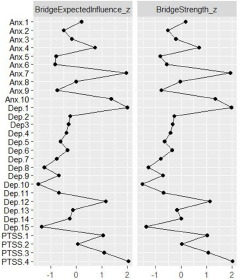
Standardized bridge strength and bridge expected influence for the Syrian sample.

For the Afghan sample, we identified the following symptoms with the highest bridge strength (BS) and bridge expected influence (BI) (Fig. 5): anger and irritability (PTSS.4) (BS = 3.185, BI = 3.167), feeling low in energy (Dep.1) (BS = 2.035, BI = 2.056) and feeling restless (Anx.10) (BS = 1.105, BI = 1.156). The symptoms with the lowest BS and BI values were feeling hopeless about the future (Dep.7) (BS = −1.554, BI = −1.918), feeling lonely (Dep.9) (BS = −1.115, BI = −0.989) and poor appetite (Dep.5) (BS = −1.020, BI = −0.897). For the Syrian sample (Fig. 6), the key bridge symptoms were anger and irritability (PTSS.4) (BS = 2.027, BI = 2.029), feeling low in energy (Dep.1) (BS = 1.977, BI = 1.980) and feeling tense (Anx.7) (BS = 1.952, BI = 1.955). The symptoms with the lowest BS and BI values were thought of ending life (Dep.10) (BS = −1.525, BI = −1.492), feeling worthless (Dep.15) (BS = −1.378, BI = −1.346) and feeling blue (Dep.8) (BS = −1.290, BI = −1.258).

### Network comparison

The network invariance test examining the network structure of Afghan and Syrian samples yielded significant results, indicating that Afghans and Syrians had different network structures in which symptoms (items of the scales) were connected differently (M = 0.204, *p* = 0.010). The strength centrality indices of the samples (calculated at the item level) were moderately correlated (*r* = 0.522). The global strength invariance was not significant (S = 0.221, *p* = 0.113), meaning that the overall connectivity of the symptoms was not significantly different. The network and global strength invariance tests were not significantly different for gender in either of the samples (M = 0.211, *p* = 0.252, S = 0.112, *p* = 0.572 for the Afghan sample; M = 0.204, *p* = 0.217, S = 0.076, *p* = 0.677 for the Syrian sample).

## Discussion

The present study provides a nuanced understanding of the central and bride symptoms across three comorbid mental disorders, namely, PTSD, depression and anxiety among culturally and linguistically distinct RAS. Overall, Afghans and Syrians differed from each other in terms of the network structure (the way that the symptoms interact) while the overall strength of the connections between the symptoms was similar in both samples. There were no differences in network structures or strength of connections between male and female participants in either sample. The current results highlighted the centrality of depression and anxiety symptoms for maintaining the overall network structure and the main bridge function of anger and irritability for driving the co-occurrence of the symptoms of these mental disorders in both samples.

For Afghan participants, feeling blue, feeling restless and spells of terror or panic were the most central symptoms, acting as the primary drivers of the overall observed network structure. For Syrians, those symptoms were worrying too much, feeling blue and feeling tense or keyed up. These findings are in line with the results of a recent study conducted with ex-Yugoslavia war survivors in Balkan and Western countries. Feeling tense or keyed up, feeling blue and spells of terror or panic were among the most central symptoms in that study as well (Schlechter, Hellmann, et al. 2021). Corroborated by the high incidence of potentially traumatic events among Afghan and Syrian RAS (Kurt *et al.*, 2022), the symptoms of PTSD appeared as bridges connecting the web of complex presentations of comorbid mental disorders, but not as central symptoms maintaining it. This might be explained by the proximal influences of post-displacement stressors on the mental health of RAS. Some studies have shown that post-displacement stressors such as structural, social and economic problems can impair mental health functioning more than traumatic experiences due to their proximal and prevailing nature (Hou *et al.*, 2020). These stressors might play a potent role in the persistence and chronicity of mental health conditions among forcibly displaced people (Behrendt *et al.*, 2023). This assumption is corroborated by recent findings on the risk factors and probable mental health problems among Afghan and Syrian RAS in Türkiye (Kurt *et al.*, 2022).

For both Afghans and Syrians, symptoms of the PTSD hyperarousal cluster (irritability, anger outburst and aggression) were found to be the main bride symptoms with the highest bridge strength and influence, followed by feeling low in energy in both samples, feeling restless among Afghans and feeling tense among Syrians. Although previous studies using the network approach yielded similar findings about the significance of hyperarousal symptoms, those studies mostly pointed out hypervigilance, characterized by exaggerated startled response, not irritability or anger (De Schryver *et al.*, 2015; Kangaslampi *et al.*, 2021; Schlechter, Hellmann, et al. 2021; Spiller *et al.*, 2017) In those studies, intrusion (e.g., re-experiencing) and avoidance symptoms of PTSD (e.g., avoidance of trauma reminders) were shown to be more central in the PTSD symptom network. It is important to note that none of the abovementioned studies examined bridge symptoms across different mental disorders. They only focused on the identification of central symptoms. Thus, the current findings provide one of the first evidence for the role of irritability and anger as a bridge symptom connecting across three most prevalent mental disorders in a sample of RAS.

One explanation for our findings on PTSD symptoms might be related to the living circumstances of Syrian and Afghan RAS in Türkiye. Forcibly displaced Syrian people are given temporary protection status, which grants them certain rights such as access to basic services, but does not guarantee exercising those rights without impediments in practice (Presidency of Migration Management, 2023). People from Afghanistan settling in Türkiye, on the other hand, need to follow regular asylum-seeking procedures following international protection law and wait until the processing of their application. Due to prolonged processing time and rejections, most Afghans live as irregular immigrants in Türkiye (Eryurt and Koç 2017). Despite differences in asylum-seeking procedures, both groups experience varying degrees of uncertainty and temporariness, which likely contribute to frustration, anger and worsening mental health issues (Celik *et al.*, 2021; Morriss *et al.*, 2023; Nickerson *et al.*, 2022). Further, forcibly displaced people often experience multiple and complex traumatic events, precipitating trauma-related psychological problems (Steel *et al.*, 2009). These experiences are closely related to anger and hyperarousal and re-experiencing symptoms among RAS (Hoffman *et al.*, 2018; Nickerson *et al.*, 2022).

The present findings have several implications for the design and delivery of psychological interventions for RAS. Overall, the results revealed that PTSD symptoms, particularly anger and irritability, might play an important role in the comorbidity between PTSD, depression and anxiety. Furthermore, symptoms of depression and anxiety emerged as the main mechanisms underpinning the maintenance of mental disorders, thus potentially leading to chronicity. Intervening in anger and irritability symptoms might help to break their rippling effects on depression and anxiety and prevent the occurrence of comorbidity. Recent evidence for psychosocial interventions for RAS with PTSD symptoms showed that trauma-focused cognitive behavioral therapy and eye movement desensitization and reprocessing provide the greatest benefits in terms of reducing PTSD symptoms among this population (Turrini *et al.*, 2021). Our results can guide further refinement of these interventions to directly target anger and irritability symptoms, thereby increasing their effectiveness. Further, research has shown that fostering the emotion regulation capacity of individuals can help reduce anger-related problems (Del Vecchio and O’Leary, 2004). Targeted psychological interventions with a component of improving anger management can be provided to RAS. Tackling depression and anxiety symptoms can also help break the vicious cycle of mental health symptoms. Feeling blue – a common central symptom, and feeling low in energy – the second key bridge symptom in both samples can be effectively targeted with behavioral activation strategies. Behavioral activation, as an evidence-based, cost-effective strategy, can be easily implemented for the treatment of depression and anxiety (Stein *et al.*, 2021). To date, no study has been conducted to test the effectiveness of behavioral activation as a stand-alone intervention for RAS. Transdiagnostic interventions tested among the forcibly displaced people usually include a behavioral activation component (Schäfer *et al.*, 2023). Yet, evidence of the effectiveness of behavioral activation for this population is almost nonexistent. Future studies can focus on testing the relative effectiveness of emotion-based strategies, behavioral activation, or both to prevent a cascade of mental health issues among forcibly displaced people. Further, psychological interventions targeting central and bridge symptoms might be beneficial for improving low treatment engagement and adherence among this population (Semmlinger and Ehring, 2022) as these interventions might accelerate symptom reduction, leading to experiencing treatment gains faster.

To our knowledge, this study was the first to apply a network approach to elucidate the relationships between the symptoms of highly prevalent comorbid mental disorders among culturally and linguistically diverse RAS in a resource-limited, protracted resettlement setting. The estimated network structures had high accuracy and stability in both samples. The sample sizes were large enough to allow for meaningful comparisons between two forcibly displaced communities in Türkiye. The present findings provided novel insights into comorbidity and relationships between the symptoms of three common mental disorders. However, due to the cross-sectional nature of our data, we could not explore the directionality between the edges. Knowing that network structures are amenable to change over time (Greene *et al.*, 2020), it is necessary to replicate our findings in a longitudinal study. Although we used validated instruments for both samples, they are not designed to detect idioms of distress. Thus, the most central and bridge symptoms may not have been captured with those instruments. Further, to ascertain the centrality of the identified symptoms, future studies should provide evidence by showing that intervening in the central symptoms leads to disruption in the network and symptom reduction. To do so, in addition to conducting effectiveness analysis, studies can demonstrate how the tested interventions change symptom structures from the pre- to post-intervention period.

To conclude, this network analysis study provided initial evidence to which key symptoms can be targeted by psychological interventions to reduce the mental health burden among culturally and linguistically diverse RAS.

## Supporting information

Kurt et al. supplementary material Kurt et al. supplementary material

## Data Availability

The data that support the findings of this study are available from the corresponding author (C.A.) on reasonable request.

## Supplementary material

The supplementary material for this article can be found at https://doi.org/10.1017/S2045796024000696.
