# Supplementary material for: Network analytical investigation of relationships between symptoms of common mental disorders among refugees and asylum seekers in Türkiye
Source: Epidemiol Psychiatr Sci. 2024 Nov 5;33:e59. doi: 10.1017/S2045796024000696 (PMC11561678; doi:10.1017/S2045796024000696)

**Supplementary Material.**


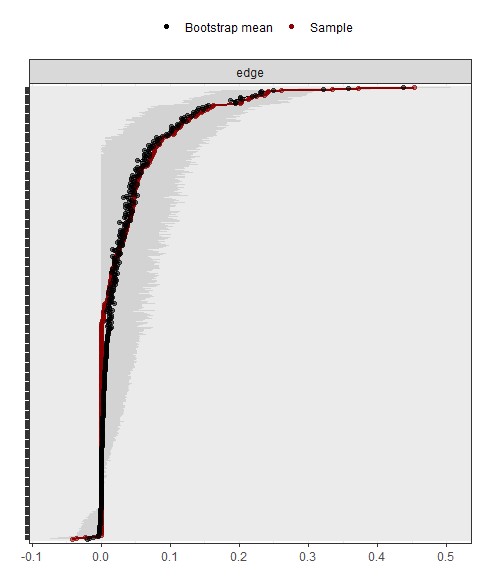
**S1.** Bootstrapped Edge Weights for the Afghan sample.


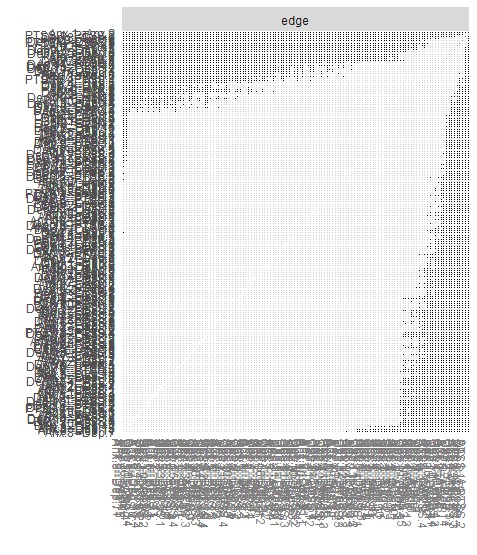
**S2.** Edge Weight Differences for the Afghan sample.


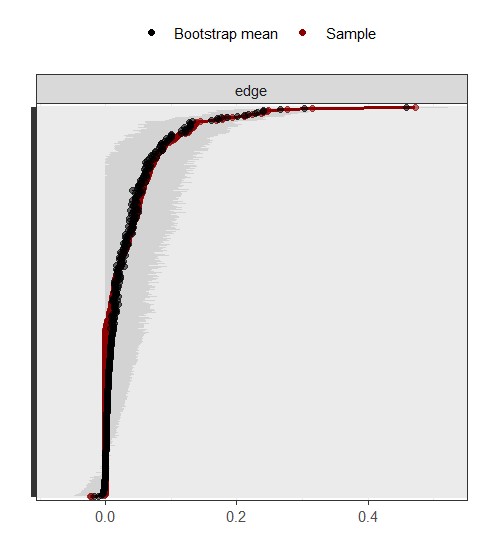
**S3.** Bootstrapped Edge Weight for the Syrian sample.

**S4.** Edge Weight Differences for the Syria sample.


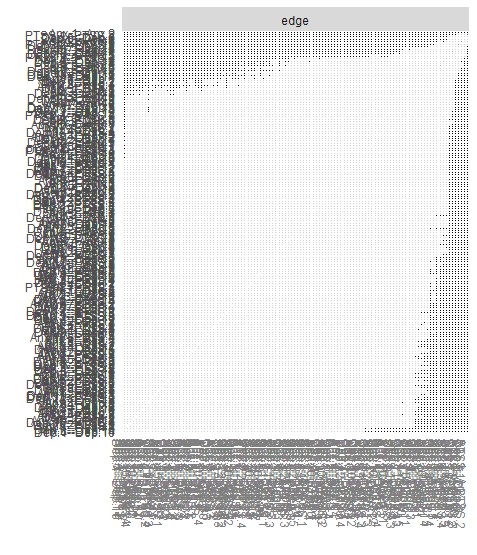

Supplement: Kurt et al. supplementary material [file S2045796024000696sup001.docx]
